# Mechanistic Studies on the Role of IL-17/NLRP3 in Arsenic-Induced Activation of Hepatic Stellate Cells Through Hepatocyte Proptosis

**DOI:** 10.3390/toxics13040287

**Published:** 2025-04-09

**Authors:** Ting Hu, Mei Chen, Sai Tian, Peng Luo, Jiangping Zhang

**Affiliations:** 1 The Key Laboratory of Environmental Pollution Monitoring and Disease Control, Ministry of Education, School of Public Health, Guizhou Medical University, Guiyang 561113, China; hutinggmc@162.com (T.H.); shawtyeveryday123@126.com (M.C.); 19985114616@163.com (S.T.); 2 Guiyang Public Health Clinical Center, Guiyang 550003, China

**Keywords:** arsenic, pyroptosis, IL-17, NLRP3, HSCs

## Abstract

Long-term exposure to arsenic, a prevalent environmental contaminant, has been implicated in the pathogenesis of various hepatic conditions. Hepatic stellate cells (HSCs) are central to the development of liver fibrosis. Recently, the involvement of interleukin-17 (IL-17) and the NOD-like receptor protein 3 (NLRP3) inflammasome in hepatic pathologies has attracted significant research interest. Hepatocyte pyroptosis, a form of programmed cell death, is a critical factor in the occurrence of inflammation. The objective of this study was to investigate the specific roles of IL-17 and NLRP3 in the arsenic-induced activation of HSCs through hepatocyte pyroptosis. We pretreated MIHA cells with MCC950 (1 and 5 μM) and secukinumab (10 and 100 nM) for 4 h, then with NaAsO_2_ (25 μM) for 24 h at 37 °C under 5% CO_2_. After incubation, the cell-culture supernatant was collected and mixed with serum-free high-glucose DMEM medium in a 1:1 ratio to prepare the conditioned medium, which was subsequently used for the culture of LX-2 cells. The results showed that exposure to NaAsO_2_ induced hepatocellular pyroptosis, which led to the release of the inflammatory cytokines IL-18 and IL-1β and subsequent activation of HSCs. Treatment with the inhibitors MCC950 and secukinumab significantly reduced the secretion of Extracellular matrix (ECM) components and attenuated HSC activation. These results demonstrate that blocking the IL-17 and NLRP3 signaling pathways significantly reduces HSC activation and attenuates hepatic fibrogenesis. These results provide novel molecular targets for the prevention and treatment of arsenic-related liver fibrosis.

## 1. Introduction

Arsenic contamination poses a significant global health challenge, particularly in developing nations, where drinking-water sources are frequently contaminated with this hazardous element. Arsenic, a well-established carcinogenic agent, is naturally abundant and exists in multiple oxidation states, presenting in organic and inorganic varieties. Its toxic effects have led to substantial global health concerns [1]. As a potent toxicant, arsenic can infiltrate the human system through multiple pathways, such as consumption of tainted food or water and inhalation of arsenic in its gaseous form. Upon entry into the body, it may impair various organ systems, resulting in severe health consequences [2]. A substantial corpus of epidemiological data has been amassed, and it has been shown that exposure to arsenic can result in the onset and progression of several types of cancers, including bladder, skin, lung, and kidney cancers. In addition to its carcinogenic properties, arsenic has been shown to have neurotoxic and reproductive toxicity [3,4,5]. The liver is a vital organ responsible for the regulation of metabolic processes, and it plays a key role in the maintenance of normal physiological functions. Liver diseases, including fibrosis, cirrhosis, and cancer, pose a significant health threat given the high morbidity and mortality rates associated with them. The economic and health burdens associated with these disorders have driven increased attention toward preventing and managing liver diseases [6]. Accumulating evidence suggests that chronic arsenic exposure contributes to hepatic injury and inflammation, which are characterized by dysregulation of liver immune homeostasis and elevated secretion of inflammatory mediators, including IL-1β and IL-6 [7,8,9]. Emerging evidence indicates that the pathological cascade of hepatic inflammation serves as a critical driver of the pathogenesis and progression of chronic liver diseases, including liver fibrosis, cirrhosis, and hepatocellular carcinoma (HCC) [10].

Liver fibrosis is defined as a reversible wound-healing response that is characterized by the accumulation of a protein called ECM following injury to the liver [11]. In cases where liver injury is acute or self-limiting, the liver can be restored; however, if the injury persists, there is a risk of inflammation and scar-tissue build-up, which can lead to cirrhosis [12]. Therefore, studying hepatic fibrosis is important for developing treatments for liver injury. It has been shown that HSCs play a key role in liver fibrosis. In normal hepatic tissue, HSCs maintain a dormant state in which they are responsible for vitamin A storage and regulation of the sinusoidal circulation. However, persistent liver damage from conditions like viral hepatitis, alcoholic liver disease, and non-alcoholic fatty liver disease triggers the activation of HSCs and their transformation into myofibroblasts. This transformation is characterized by enhanced cell proliferation and migration, as well as the excessive synthesis of ECM components, including type I, III, and IV collagen, as well as fibronectin, laminin, and proteoglycans, ultimately leading to the development of liver fibrosis [13].

A key driver of hepatic fibrogenesis is HSC activation, which occurs in two distinct stages: initiation and perpetuation. In the initiation stage, persistent hepatic damage stimulates HSCs via the secretion of pro-inflammatory mediators, including IL-1β [14]. In the perpetuation phase, activated HSCs maintain their activated state through autocrine and paracrine mechanisms. For example, TGF-β1 secreted by HSCs not only promotes HSC proliferation but also further stimulates the synthesis of ECM [15]. In a healthy liver, ECM forms a highly dynamic structure characterized by a tightly regulated equilibrium between its synthesis and degradation. However, in the context of chronic liver injury, the synthesis of ECM markedly exceeds its degradation [16]. This imbalance causes gradual thickening of fibrotic septa and collagen cross-linking, driving hepatic fibrogenesis. In addition, the progression of liver fibrosis is exacerbated by alterations in the composition of the ECM, which directly promote fibrogenesis [17]. Chronic inflammation is a significant driving factor in liver fibrosis. Inflammatory mediators, such as TNF-α and IL-6, activate HSCs, the primary effector cells in liver fibrosis. Activated HSCs not only synthesize large amounts of ECM but also express pro-inflammatory mediators and α-smooth muscle actin (α-SMA) [18]. Moreover, Th17 cells regulate HSC activation and fibrosis progression by releasing cytokines such as IL-17. In addition, inflammatory substances produced by HSCs further act on hepatocytes, contributing to the persistence of liver inflammation [13,14]. Based on the mechanisms underlying the development of liver fibrosis, current treatments for liver fibrosis primarily focus on targeting the inhibition of HSC activation and immune modulation [13].

IL-17, a pro-inflammatory mediator secreted by Th17 cells, plays a significant role in various inflammatory and autoimmune disorders [19]. Studies have demonstrated that IL-17 activates hematopoietic stem cells by upregulating TGF-β1 [20]. Additionally, IL-17 activates hematopoietic stem cells by activating the inflammatory vesicle NLRP3, which leads to apoptosis in the liver parenchyma cells [21]. Pyroptosis, a programmed cell-death mechanism, is characterized by nuclear condensation, DNA fragmentation, plasma-membrane disruption, and the generation of membrane-bound vesicles that discharge pro-inflammatory mediators [22]. Pyroptosis represents a distinct form of programmed cell death mediated by inflammasome assembly, caspase-cascade activation, and subsequent release of inflammatory mediators. This process is initiated by the assembly of ASCs (apoptotic-speck-like proteins) containing caspase-recruitment domains, NOD-like receptors, and caspase-1 proteins. These components are found within inflammasome-activated caspase-1 proteins, such as the most typical NLRP3 inflammasome [23]. The NLRP3 inflammasome, a multiprotein complex comprising NLRP3, ASC, and caspase-1, is triggered by diverse stimuli including microbial pathogens and endogenous danger signals. Upon activation, it enhances inflammatory signaling through the proteolytic maturation and release of IL-1β and IL-18 [24].

Current research suggests potential roles of IL-17 and NLRP3 inflammasome pathways in arsenic-triggered hepatocyte pyroptosis and HSC activation; however, the precise molecular mechanisms remain largely unexplored. We propose that prolonged arsenic exposure induces hepatocyte pyroptosis through IL-17/NLRP3 signaling; this results in the release of pro-inflammatory cytokines and subsequent activation of HSCs, which promote the development of liver fibrosis. Grounded in the “inflammation–fibrosis” paradigm and the mechanistic framework of pyroptosis, this study aims to elucidate the molecular pathways linking IL-17/NLRP3-mediated hepatocyte pyroptosis to HSC activation, thus revealing potential therapeutic targets for treating arsenic-related hepatic fibrosis.

## 2. Material and Methods

### 2.1. Instruments and Reagents

The reagents and instruments used in the experiments were as follows: sodium arsenite (Sigma, St. Louis, MO, USA); fetal bovine serum, DMEM, and 1640 medium (purchased from Gibico, Grand Island, NY, USA); dimethyl sulfoxide (DMSO) and a BCA protein-quantification kit (purchased from Solarbio, Beijing, China); Trizol reagent (purchased from Invitrogen, Carlsbad, CA, USA); a TB Green fluorescent quantitative PCR kit (purchased from TakaraBiomedical Technology, Dalian, China); SYBR Green fluorescent quantitative reagent (purchased from Vazyme Biotech, Nanjing, China); and qRT-PCR primers (synthesized by Sangon Biotech, Shanghai, China). The experimental instruments included a clean bench (Suzhou Yida Purification Steel Structure Co., Ltd., Suzhou, China), a Max200 microplate reader (Bio-Tek, Instruments, Winooski, VT, USA), electrophoresis and transfer equipment for western blotting, a gel imaging system (Bio-Rad, Laboratories, Hercules, CA, USA), a CFX-96 PCR instrument (Bio-Rad, Laboratories, Hercules, CA, USA), and ELISA kits (purchased from Jianglai, Shanghai, China; Taipei, Taiwan). Additionally, MCC950 and secukinumab were purchased from MCE, and the CCK8 assay kit (purchased from UElandy, Shenzhen, China).

### 2.2. Cell Culture

MIHA cells (Fenghui Biologicals, Changsha, China) and LX-2 cells (Cell Bank of Chinese Academy of Sciences, Shanghai, Chaina) were cultured in RPMI 1640 and high-glucose DMEM media, respectively. The culture media were supplemented with 10% FBS for MIHA cells and 2% FBS for LX-2 cells, along with 100 μg/mL streptomycin and 100 IU/mL penicillin.

### 2.3. NaAsO_2_ Treated Cell

MIHA cells were inoculated into 96-well plates at 8 × 10^3^ cells/well and cultured for 24 h. After 24 h, MIHA cells were stained with NaAsO_2_ at concentrations of 0, 2.5, 5, 1, 20, 40, 80, and 160 μM for 24 h. The IC50 value of NaAsO_2_ was calculated and used in the subsequent experiments. Using IC50 values as a reference, MIHA cells were treated with 6.25, 12.5, and 25 μM (1/16, 1/8, and 1/4 IC50, respectively) for 24 h before collection for further analysis.

### 2.4. Inhibitor Treatment of Cells

NLRP3 inflammasome activation requires two key stages: priming and assembly.

MCC950, a specific NLRP3 inhibitor, targets the assembly step, whereas secukinumab, a humanized monoclonal antibody, binds IL-17A to block its interaction with its receptor and suppress inflammation. Here, MCC950 and secukinumab were used as inhibitors of NLRP3 and IL-17 (MedChemExpress, Monmouth Junction, NJ, USA), respectively, for the study of hepatic stellate cell-activation mechanisms.

When cell confluence reached 50–60%, MIHA cells were pretreated with (1, 5 μM) MCC950 or (10, 100 nM) secukinumab for 4 h in 1640 medium supplemented with 2% FBS. Following pretreatment, the cells were treated with 25 μM NaAsO_2_ and incubated at 37 °C with 5% CO_2_ for 24 h. After 24 h, the cell-culture supernatant was collected into 15 mL centrifuge tubes for the preparation of conditioned medium for LX-2 cell culture. The cells were harvested for the extraction of relevant genes and proteins.

### 2.5. Conditioned Culture

After collection of the culture supernatant, the medium was centrifuged at 3000 rpm/20 min. The resulting supernatant was mixed with serum-free high-glucose DMEM medium at a ratio of 1:1 to prepare conditioned medium for LX-2 cell culture [25].

### 2.6. CCK8 for Cell Viability

MIHA cells were treated as described in Section 2.3 and Section 2.4. Following treatment, CCK8 reagent was added and cells were incubated at 37 °C for 2 h. Absorbance was measured at 450 nm using a microplate reader (Bio-Tek Instruments, Winooski, VT, USA).

### 2.7. Western Blot

Cells were gently scraped and centrifuged at 1000 rpm for 10 min. Afterward, the cells were resuspended in a mixture of lysate (RIPA) and protease inhibitor (PMSF), with the dosage of the latter adjusted according to the established protocol. Cells were denatured by heating at 100 °C for 10 min. The separated proteins were transferred by wet electrophoresis to polyvinylidene difluoride. Proteins were resolved on 15% and 8% SDS-PAGE gels and then transferred to PVDF membranes (Merck Millipore, ISEQ00010, Billerica, MA, USA) via wet transfer. The membrane was then blocked with TBST-configured 2% bovine serum protein at room temperature for 2 h. A mixture of diluted pro-caspase-1 (Abcom, ab179515, 1:1000, Cambridge, UK), cleaved caspase-1 (Abclonal, A23429, 1:1000, Wuhan, China), NLRP3 (Abclonal, A12694, 1:1000, Wuhan, China), TGFβ-1 (Proteintech, 26155-1-AP, 1:1000, Wuhan, China), α-SMA (Bioss bsm-33187 M, 1:1000), and GAPDH (BOSTER, BA1050, 1:10,000, Wuhan, China) was used as a loading control. Membranes were incubated overnight, and this step was followed by a 1 h incubation with goat anti-rabbit IgG (Proteintech, SA00013-2, 1:10,000) or goat anti-mouse IgG (Proteintech, SA00013-1, 1:10,000, Wuhan, China) secondary antibodies at room temperature. The visualization of the bands was carried out using an ultra-sensitive ECL chemiluminescent ready-to-use substrate (Beyotime Biotechnology, Shanghai, China). The gray values of the bands were then analyzed using Image J (Version 1.54, National Institutes of Health, Bethesda, MD, USA). GAPDH was used as a control to ensure homogeneity of egg volume and to compensate for variability.

### 2.8. qPCR

Total RNA was extracted from MIHA and LX-2 cells using TRIzol reagent (Invitrogen, Carlsbad, CA, USA), with 2 μg and 1 μg, respectively, quantified for subsequent analysis. RNA purity was evaluated by spectrophotometry (260/280 nm ratio: 1.8–2.1). cDNA was synthesized using a TakaRa kit, after which qRT-PCR was conducted on a Bio-Rad CFX96 system. GAPDH served as the internal control, and primers (Table 1) were designed and synthesized by Shanghai Sangyo. Relative gene expression was calculated using the 2ΔΔCt method.

### 2.9. ELISA

Cell-culture supernatants were centrifuged at 3000 rpm for 20 min and stored at 4 °C or −20 °C for further analysis. IL-1β, IL-18, and IL-17 levels in MIHA cell supernatants were measured using ELISA, while ECM components (HA, LN, PC-III, and COL-IV) in LX-2 cell supernatants were also quantified.

### 2.10. Analyze Statistics

Statistical analyses were conducted with GraphPad Prism 8.0. Comparisons between two groups were performed using an independent samples *t*-test, while differences among multiple groups were examined through one-way ANOVA. Dunnett’s test was applied to compare each experimental group with the control group. Data are presented as mean ± standard deviation (*x* ± *s*). A significance level of α = 0.05 was adopted, with *p* < 0.05 considered statistically significant.

## 3. Results

### 3.1. Effect of Different Concentrations of Sodium Arsenite on MIHA Cell Viability

**Figure 1:**
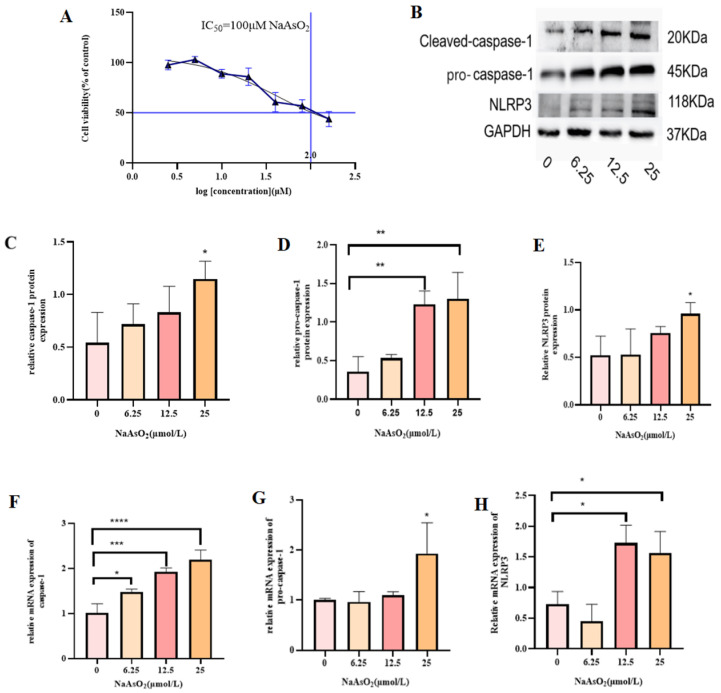
(**A**) MIHA cell viability following treatment with varying NaAsO_2_ concentrations (*n* = 12). (**B**) Representative immunoblots showing NLRP3, pro-caspase-1, and caspase-1 protein bands. (**C**–**E**) Protein-expression levels of NLRP3, pro-caspase-1, and caspase-1 in MIHA cells under different NaAsO_2_ concentrations. (**F**–**H**) mRNA-expression levels of NLRP3, pro-caspase-1, and caspase-1 in MIHA cells treated with NaAsO_2_. (* *p* < 0.05, ** *p* < 0.01, *** *p* < 0.001, **** *p* < 0.0001, indicating statistically significant differences).

MIHA cells were exposed to varying concentrations of NaAsO_2_ (0, 2.5, 10, 20, 40, 8, and 160 μmol/L), and by nonlinear fitting of the absorbance, it was determined that there was no inhibitory effect on MIHA cells at a concentration of 2.5 μmol/L. However, with increases in NaAsO_2_ concentration, the inhibitory effect on MIHA cells was shown to be dose-dependent. The IC_50_ value of NaAsO_2_ in MIHA cells was calculated to be 100 μmol/L (Figure 1A). For further experiments, sub-cytotoxic doses of 6.25, 12.5, and 25 μmol/L, representing 1/16, 1/8, and 1/4 of the IC_50_, respectively, were selected.

### 3.2. NaAsO_2_ Induces Inflammation and Proptosis in MIHA Cells

As shown in Figure 1, increasing NaAsO_2_ concentrations (6.2, 12.5, and 25 μmol/L) upregulated the expression of NLRP3, pro-caspase-1, and caspase-1 in treated MIHA cells compared to the control group. Levels of both mRNAs and proteins exhibited a dose-dependent increase, with the most significant effects observed at 25 μmol/L (*p* < 0.05). These findings indicate that NaAsO_2_ induces an inflammatory response in MIHA cells, promoting NLRP3 inflammasome activation and caspase-1-mediated hepatocyte death. Based on these results, 25 μmol/L was selected as the optimal dose for further experiments.

### 3.3. Pre-Treatment of NaAsO_2_ with Different Concentrations of the Inhibitors MCC950 and Secukinumab Induced the Pyroptotic Effects of Related Genes and Proteins in MIHA Cells

**Figure 2:**
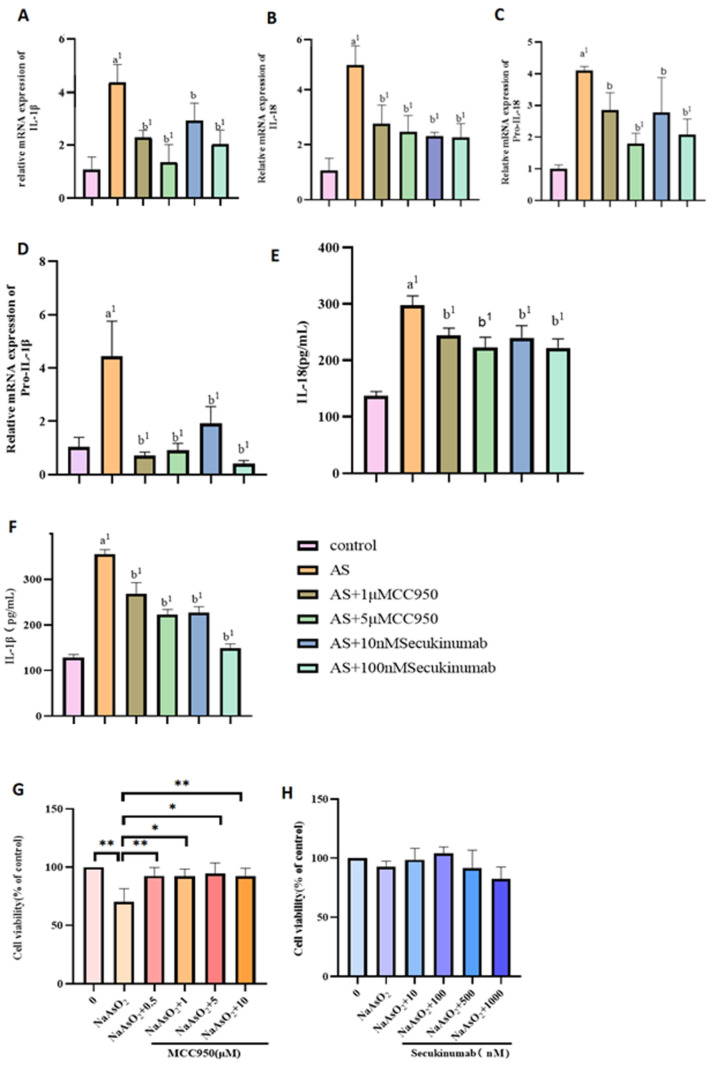
(**A**–**D**) PCR was used to detect the mRNA-expression levels of pro-IL-18, pro-IL-1β, IL-18, and IL-1β in MIHA cells treated with different concentrations of the inhibitors MCC950 and secukinumab. (**E**,**F**) ELISA was used to detect the expression of IL-18 and IL-1β proteins in MIHA cell-culture supernatant treated with the inhibitors MCC950 and secukinumab. (**G**,**H**) The effects of MCC950 (NLRP3 inhibitor) and secukinumab (IL-17 inhibitor) on the viability of MIHA cells; a: the NaAsO_2_-exposed group was compared with the control group; b: inhibitor group compared with NaAsO_2_ group. With *p* < 0.05; the difference was statistically significant. (* *p* < 0.05, ** *p* < 0.01, ^b^ *p* < 0.05; ^a1,b1^ *p* < 0.01).

As shown in Figure 2G,H, MIHA cells were pretreated with varying concentrations of MCC950 and secukinumab for 4 h after NaAsO_2_ exposure, and this step was followed by a 24 h incubation. Cell viability was assessed using the CCK8 assay, which revealed that 1 μM and 5 μM MCC950, combined with 10 nM and 100 nM secukinumab, respectively, significantly affected cell viability post-NaAsO_2_ treatment. These concentrations were therefore selected for subsequent experiments.

**Figure 3:**
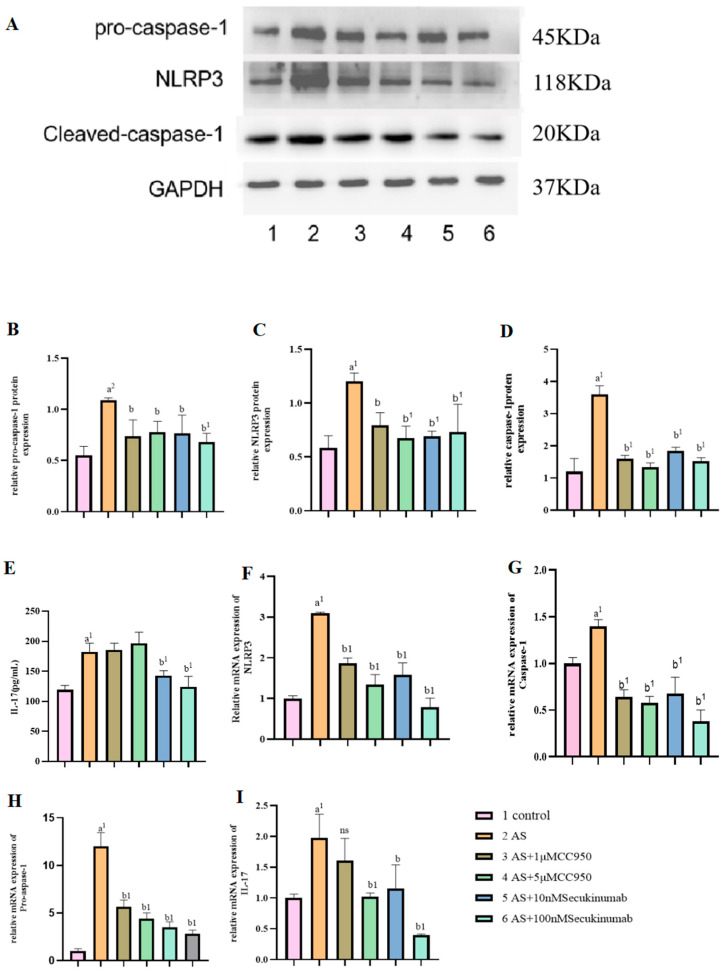
(**A**) Bands visualized for pro-caspase-1, caspase-1, and NLRP3 proteins. (**B**–**D**) Effect of pretreatment with MCC950 and secukinumab on the expression of proteins associated with inflammation and pyroptosis in MIHA cells stained with NaAsO_2_. (**E**) ELISA assay of expression levels of the inflammatory factor IL-17 in MIHA cell-culture supernatant. (**F**–**I**) PCR for pro-caspase-1, caspase-1, NLRP3, and IL-17 mRNA expression in MIHA cells. a: NaAsO_2_ group vs. control group (^a1^ *p* < 0.01), b: NaAsO_2_ group vs. inhibitor group (^b^ *p* < 0.05, ^b1^ *p* < 0.01). ns: Compared with the NaAsO_2_ group, *p* > 0.05, the difference was not statistically significant. At *p* < 0.05, differences were statistically significant.

Following NaAsO_2_ exposure, mRNA levels of NLRP3, pro-caspase-1, caspase-1, pro-IL-18, pro-IL-1β, IL-18, IL-1β, and IL-17 were elevated in the exposed groups compared to the control group 3, indicating NLRP3 and caspase-1 activation. In MCC950-treated cells, expression of pro-caspase-1, caspase-1, pro-IL-18, pro-IL-1β, IL-18, and IL-1β decreased, while IL-17 levels remained unchanged. Similarly, secukinumab treatment reduced NLRP3, pro-caspase-1, caspase-1, pro-IL-18, pro-IL-1β, IL-18, and IL-1β at both the protein and mRNA levels. ELISA analysis of culture serum further confirmed decreased levels of IL-18, IL-1β, and IL-17 in the MCC950- and secukinumab-treated groups, with no significant change in IL-17 expression observed in the MCC950 group (e.g., Figure 2 and Figure 3).

### 3.4. Effect of Conditioned Medium on LX-2 Cell Activation

**Figure 4:**
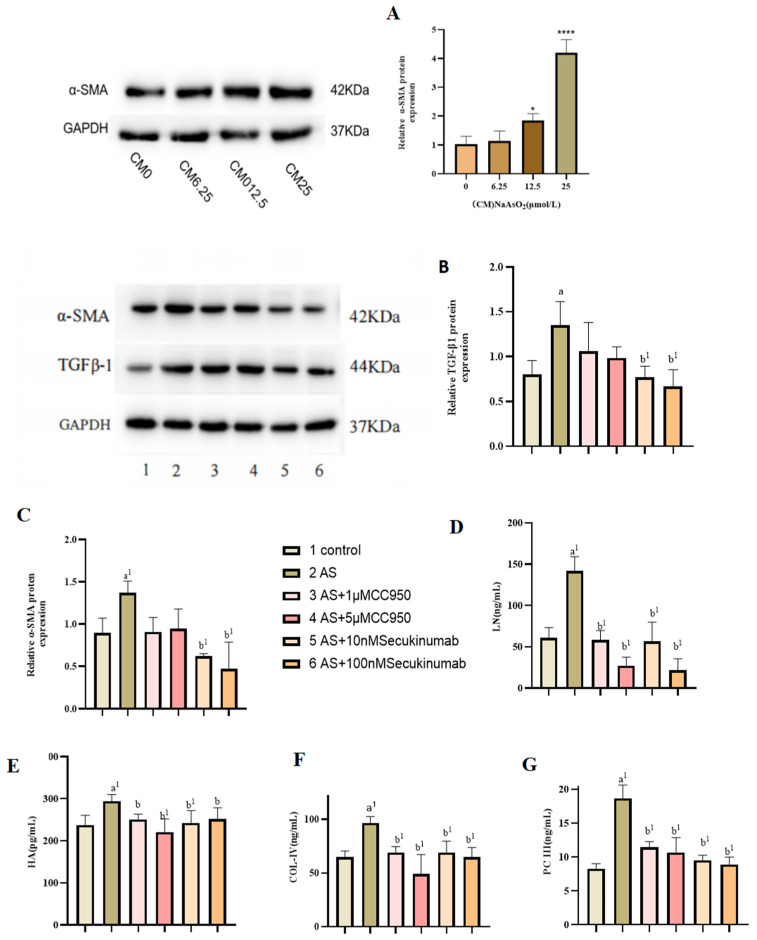
(**A**) Effect of conditioned medium (supernatant from MIHA cells treated with varying NaAsO_2_ concentrations) on α-SMA protein levels in LX-2 cells. (**B**,**C**) Impact of conditioned medium on the expression of the activation-related proteins TGFβ-1 and α-SMA in LX-2 cells. (**D**–**G**) ELISA analysis of changes in the extracellular matrix (ECM) in the culture supernatant of LX-2 cells treated with conditioned medium. Comparisons include: a: NaAsO_2_-exposed group versus control group; b: inhibitor-treated group versus NaAsO_2_-exposed group. (^a,b^ *p* < 0.05, ^a1,b1^ *p* < 0.01; * *p* < 0.05. **** *p* < 0.001, *p* < 0.05 were statistically significant).

Compared with the control group, the expression of α-SMA in LX-2 cells was gradually increased in a dose-dependent manner after treatment with MIHA cell supernatant after NaAsO_2_ treatment (*p* < 0.05). These results indicated that the conditioned medium could successfully activate LX-2 cells and induce the activation of hepatic stellate cells (e.g., Figure 4A). As shown in (e.g., Figure 4B,C) reatment of LX-2 cells with MIHA cell-culture supernatants resulted in increased expression of α-SMA and TGF-β1 in the NaAsO_2_-exposed group. However, following treatment with MCC950 and secukinumab at varying concentrations, the expression of α-SMA and TGF-β1 in hepatic stellate cells was significantly reduced, with a more pronounced decrease observed after IL-17 inhibition. Furthermore, ECM expression was detected in the culture medium of LX-2 cells, revealing that the levels of ECM components, including HA, LN, PC-III, and COL-IV, were elevated in the treated group. However, these levels were found to decrease following various inhibitory treatments, suggesting that treatment of the conditioned medium induced the activation of LX-2 cells, leading to an increase in collagen formation, α-smooth muscle actin expression, and ECM deposition (e.g., Figure 4D–G).

## 4. Discussion

Our results indicate that arsenic exposure triggers NLRP3 inflammasome activation, leading to the conversion of pro-caspase-1 into its active form, caspase-1. This process promotes hepatocyte pyroptosis and ultimately activates HSCs. Importantly, the pro-inflammatory cytokine IL-17 is a key mediator in this pathway. However, the precise molecular mechanisms underlying this cascade, especially the complex interplay between IL-17 and the NLRP3/caspase-1 signaling axis, require further in-depth investigation.

Despite the well-documented toxicity of arsenic, its environmental-exposure pathways (including water, air, and food contamination) and its extensive utilization in industry and agriculture (e.g., in pesticides and herbicides) have yet to be effectively regulated. As a prototypical persistent, bioaccumulative, and carcinogenic substance, arsenic’s enduring presence in the environment continues to pose significant and far-reaching threats to human health. Although numerous in vivo and in vitro studies, as well as epidemiological investigations, have elucidated the toxic effects of arsenic exposure, the precise molecular mechanisms underlying arsenic toxicity remain incompletely understood [26]. Arsenic compounds, in diverse chemical forms, can infiltrate the human body via ingestion, inhalation, or dermal absorption, accumulating in various organs and inducing systemic toxicity. Notably, the kidneys, liver, lungs, skin, nervous system, and reproductive system are particularly susceptible to arsenic exposure, which may result in varying degrees of pathological damage, including tissue inflammation, fibrosis, functional impairment, and even carcinogenesis. The liver, as a primary target organ for arsenic toxicity, plays a central role in arsenic metabolism [27,28].

Current research on the mechanisms of arsenic toxicity has extensively utilized various animal models and cellular experimental systems, yielding significant advancements. Studies have demonstrated that the toxic effects of arsenic exposure are mediated by multiple molecular mechanisms, primarily including oxidative stress [29,30], inflammatory responses [31], alterations to signaling pathways [32], and epigenetic alterations (such as DNA methylation) [33]. Research has shown that arsenic-induced liver injury involves multiple pathophysiological processes, including oxidative damage to DNA, enhanced apoptotic resistance, abnormal cell proliferation, alterations in DNA-methylation patterns, and genomic instability [34,35]. These molecular alterations collectively drive the initiation and progression of arsenic-induced liver pathologies. Numerous studies have utilized in vitro hepatocyte culture models to investigate the molecular mechanisms underlying arsenic-induced liver injury [36,37]; the goals of such research align closely with the objectives of the present study. Building upon this foundation, we established a model of the indirect interaction between hepatocytes and HSCs through arsenic exposure in a co-culture system, aiming to systematically explore the potential relationship between arsenic exposure and HSC activation, as well as the underlying molecular mechanisms.

In our study, we employed an in vitro MIHA cell model in which the cells were exposed to 25 μM NaAsO_2_, Through molecular biology assays, we observed the occurrence of characteristic pyroptosis in hepatocytes. This result aligns with the findings of Zhang et al. [38], who also observed arsenic-induced hepatocyte pyroptosis in an ex vivo animal model. The critical prerequisite for hepatocyte pyroptosis is the activation of the NLRP3 inflammasome and the subsequent release of its downstream effector molecule, caspase-1. Our study revealed that under NaAsO_2_ exposure, hepatocytes exhibited not only a significant upregulation of NLRP3 expression but also a marked increase in both the gene and protein levels of IL-17. These findings indicate that, in arsenic-induced hepatic inflammation, the activation of the NLRP3 inflammasome is accompanied by a significant enhancement in the expression of the inflammatory cytokine IL-17. To further validate the role of IL-17 in the pyroptosis process, we treated arsenic-exposed hepatocytes with secukinumab, a specific IL-17 inhibitor. The results revealed a significant reduction in the expression levels of both NLRP3 and caspase-1. This discovery suggests that IL-17 may participate in arsenic-induced hepatocyte pyroptosis by modulating the NLRP3/caspase-1 pathway. The findings of Li et al. [39] in their study on sinusitis further support this mechanism, as they observed a significant upregulation of NLRP3 and caspase-1 expression in nasal mucosal epithelial cells following treatment with an IL-17 agonist. Similarly, in arsenic-exposed hepatocyte cultures, studies have shown that treatment with 4 μM NaAsO_2_ for 48 h significantly upregulates the expression of NLRP3 and caspase-1 in hepatocytes. These results collectively indicate that arsenic exposure indeed induces hepatocyte pyroptosis and that this process may be associated with the regulation of IL-17 expression.

To date, four primary signaling pathways have been implicated in the regulation of pyroptosis: the canonical inflammasome pathway, the non-canonical inflammasome pathway, the caspase-mediated pathway, and the granzyme-dependent pathway [40]. Among these, the canonical inflammasome pathway is the earliest elucidated mechanism for pyroptosis regulation, which triggers inflammatory responses through the activation of inflammatory caspases (e.g., caspase-1). The activation of caspases typically occurs in immune cells or inflammatory cells and represents one of the core molecular events in pyroptosis [41]. In the canonical pyroptosis pathway, caspase-1 is activated by multiprotein complexes known as inflammasomes, with NLRP3 being the most prevalent. NLRP3 inflammasomes comprise intracellular pattern-recognition receptors (PRRs), apoptosis-associated speck-like proteins (ASCs), and inflammatory caspases [42,43]. Upon PRR stimulation, pro-caspase-1 assembles into caspase-1-dependent inflammasomes, leading to its self-activation. Active caspase-1 then cleaves inactive precursors of IL-1β and IL-18. This ultimately leads to an inflammatory response and cellular pyroptosis [44]. This mechanism is highly consistent with our research findings. Using WB and PCR analyses, we detected a marked increase in IL-18 and IL-1β expression in liver cells after arsenic exposure. Importantly, inhibition of IL-17 and NLRP3 significantly reduced IL-18 and IL-1β levels, further validating the critical role of the NLRP3/caspase-1/IL-17 signaling axis in arsenic-triggered hepatocyte pyroptosis.

Arsenic exposure not only triggers cell pyroptosis but also disrupts helper T cell (Th) differentiation, particularly affecting the balance between Th1, Th2, and Th17 subsets, as has been shown in the lungs and spleen of mice [44]. IL-17, a key inflammatory cytokine produced by Th17 cells, plays a critical role in autoimmune and inflammatory diseases [45]. In the liver, IL-17 is often highly expressed in both parenchymal and non-parenchymal cells following external stimuli. Studies have shown that in liver-fibrosis models, IL-17 upregulates TGF-β1 and TGF-βRII expression in HSCs, activating the TGF-β1 signaling pathway. This enhances HSCs’ responsiveness to TGF-β, promoting their activation and accelerating fibrosis progression [46]. In our previous studies, we observed that arsenic exposure led to an increase in IL-17 expression in hepatocytes. Concurrently, existing reports suggest that the development of liver fibrosis may be associated with pyroptosis in parenchymal cells. Specifically, the NLRP3-activated pyroptosome, caspase-1, cleaves the precursors of IL-18 and IL-1β, releasing their mature forms. These cytokines, in turn, activate HSCs, thereby promoting the progression of fibrosis. This connection underscores the potential interplay between arsenic-induced IL-17 upregulation, hepatocyte pyroptosis, and the activation of fibrotic pathways in the liver [47]. This mechanism highlights the critical role of IL-17 in liver fibrosis. Consequently, we proceeded with the preparation of conditioned medium, specifically, the culture medium of treated hepatocytes, and subsequently used it for the treatment of LX-2 cells. Our findings revealed that the expression of α-SMA and TGF-β1 was augmented in LX-2 cells in the arsenic-treated group. Given this observation, we hypothesized that LX-2 cells might undergo activation following treatment. To further substantiate this hypothesis, we detected the expression levels of ECM in the culture supernatant by ELISA and found that the expression levels of HA, LN, PC-III, and COL-IV were elevated to different degrees, indicating that LX-2 cells were activated, but whether the cells were activated by the arsenic in the conditioned medium or by IL-17/NLRP3/caspase-1 was not clear. Subsequent ELISA assay analysis of the culture media revealed elevated levels of HA, LN, PC-III, and COL-IV, suggesting LX-2 cell activation. However, the specific mechanism by which LX-2 cells are activated remains unclear. Subsequent experiments will involve direct treatment of LX-2 cells with IL-17 proteins to ascertain whether the IL-17/NLRP3/caspase-1 pathway can be activated by the relevant proteins. However, the present study revealed a significant elevation in IL-17 levels in MIHA cells following arsenic treatment. When this result is considered in conjunction with related studies, it leads to the hypothesis that the activation of LX-2 cells is associated with elevated IL-17 expression.

We assessed α-SMA and TGF-β1 expression in LX-2 cells by inhibiting IL-17 and NLRP3. It was found that after IL-17 was inhibited, the protein expression of α-SMA and TGF-β1 significantly decreased and the secretion of ECM was also reduced accordingly. These findings suggest that HSC activation may be closely linked to elevated IL-17 levels. Furthermore, IL-17 plays a role in regulating hepatocyte pyroptosis. Inhibition of IL-17 significantly reduced NLRP3 expression in hepatocytes, indicating that IL-17 may indirectly contribute to HSC activation by modulating liver parenchymal cell pyroptosis. Previous studies have also established a connection between IL-17 expression and hepatic stellate cell activation [48,49,50], which further supports our hypothesis. This further corroborates our hypothesis. However, following the inhibition of NLRP3, levels of the activation markers of HSCs did not significantly decrease. This suggests that while inhibiting NLRP3 can effectively suppress the pyroptosis process in hepatocytes, the pro-inflammatory factor IL-17, produced by liver parenchymal cells exposed to NaAsO_2_, may still indirectly activate HSCs by regulating the expression of the inflammatory factors IL-18 and IL-1β. Additionally, residual NaAsO_2_ in the conditioned medium might directly act on LX-2 cells, activating HSCs through inflammation-related pathways. It is evident that the mechanism by which arsenic regulates HSC activation involves multiple signaling pathways and complex interactions among different cell types within the liver. The specific molecular mechanisms underlying these processes warrant further in-depth research and exploration.

In this study, we treated hepatocytes with arsenic and then applied the conditioned medium to HSCs. This approach confirmed that arsenic exposure induces inflammatory responses in hepatocytes, activates caspase-1, and leads to hepatocyte pyroptosis, with these steps accompanied by elevated levels of the inflammatory factor IL-17. By inhibiting the expression of IL-17 and NLRP3, we further validated the relationship between arsenic exposure and HSC activation, revealing that arsenic exposure can activate HSCs through both direct and indirect pathways. However, this study only preliminarily demonstrated that HSC activation may be associated with hepatocyte pyroptosis and the expression of IL-17 in liver inflammation, while the specific mechanisms remain incompletely understood. In follow-up research, we plan to directly treat LX-2 cells with an IL-17 agonist and combine this treatment with inhibitor experiments to further verify whether LX-2 cell activation is indeed related to IL-17, thereby deepening our understanding of the underlying molecular mechanisms.

## 5. Conclusions

**Figure 5:**
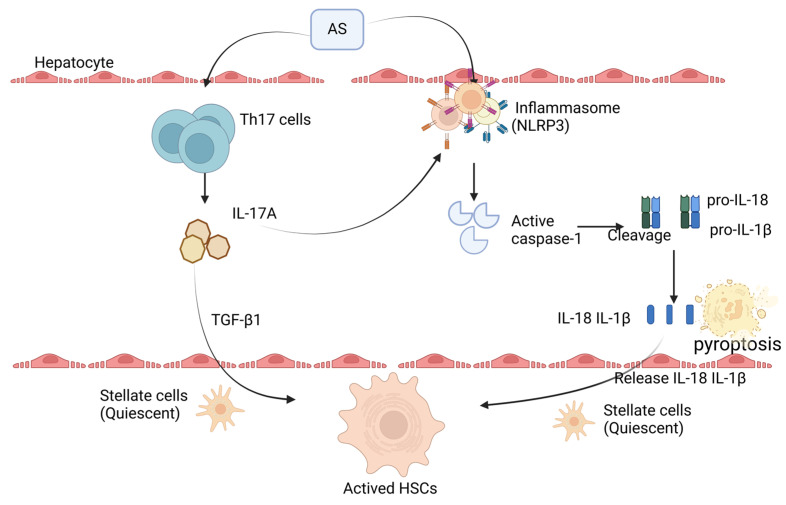
Arsenic exposure triggers hepatic inflammation, activating the NLRP3 inflammasome. This activation drives the conversion of pro-caspase-1 into active caspase-1, which processes pro-IL-18 and pro-IL-1β into their mature forms, IL-18 and IL-1β, enabling their secretion. These cytokines induce hepatocyte pyroptosis and activate HSCs, leading to excessive extracellular matrix (ECM) deposition. Furthermore, arsenic exposure stimulates hepatocytes to release IL-17, a pro-inflammatory cytokine that can either indirectly activate HSCs by regulating inflammatory mediators or directly promote HSC activation. These findings underscore the complex interplay between IL-17, inflammasome activation, pyroptosis, and HSC activation.

In summary, this study, through in vitro cell culture combined with NaAsO_2_-exposure experiments, found that arsenic exposure can induce inflammatory responses and pyroptosis in hepatocytes, responses that were accompanied by high expression of IL-17 and NLRP3. Further, after preparing conditioned medium from NaAsO_2_-treated MIHA cells and applying it to LX-2 cells, we observed significant activation of LX-2 cells and a marked increase in the expression of ECM-related components. This indicates that arsenic exposure may indirectly promote the activation of HSCs by inducing hepatocyte pyroptosis and the high expression of IL-17. Inhibition of IL-17 and NLRP3 significantly reduced the expression of pyroptosis-related proteins, including caspase-1, IL-18, and IL-1β, as well as collagen and other markers of HSC activation, in LX-2 cells. These findings offer novel experimental evidence for the molecular mechanisms underlying arsenic-induced liver fibrosis, indicating that targeting IL-17 and NLRP3 can effectively suppress HSC activation (e.g., Figure 5).

## Figures and Tables

**Table 1:** PCR primer sequences.

pro-caspase-1F	TGAAGGACAAACCGAAGG
pro-caspase-1R	GAAGAGCAGAAAGCGATA
pro-IL-18F	CATTGACCAAGGAAATCGGCC
pro-IL-18R	TAAATATGGTCCGGGGTGCA
pro-IL-1βF	GCTTGGTGATGTCTGGTCCA
pro-IL-1βR	TCAACACGCAGGACAGGTAC
IL-18 F	ATCGCTTCCTCTCGCAACAA
IL-18 R	TCCAGGTTTTCATCATCTTCAGC
IL-1beta F	ATCAGCACCTCTCAAGCAG
IL-1beta R	AGTCCACATTCAGCACAGG
NLRP3 F	GACCATCCTCGGCATGT
NLRP3 R	CACGATCCAGCAGACCA
IL-17F	AGATTACTACAACCGATCCACCT
IL-17R	GGGGACAGAGTTCATGTGGTA
Caspase-1F	GAAAAGCCATGGCCGACAGA
Caspase-1R	GCCCCTTTCATGGGTGAAGG

## Data Availability

The data presented in this study is available on request from the corresponding authors.
